# Factors associated with the elevated percentage of CD4CD69 T cells in maintained hemodialysis patients

**DOI:** 10.1080/0886022X.2017.1349672

**Published:** 2017-07-20

**Authors:** Rongyi Chen, Fangfang Xiang, Jiachang Hu, Xuesen Cao, Xiao Tan, Ping Jia, Ting Zhang, Nana Song, Yi Fang, Xiaoqiang Ding, Jianzhou Zou

**Affiliations:** a Division of Nephrology, Zhongshan Hospital, Shanghai Medical College, Fudan University, Shanghai, China;; b Shanghai Institute of Kidney and Dialysis, Shanghai, China;; c Key Laboratory of Kidney and Blood Purification of Shanghai, Shanghai, China

**Keywords:** CD4 T cell, CD69, hemodialysis, total protein, magnesium, transferrin

## Abstract

**Background:** CD4 T-cell abnormality, influencing the outcome of the maintained hemodialysis (MHD), is common in patients on dialysis. We try to find out factors associated with the activated CD4 T cells, CD4CD69 T cells, to improve the dialysis quality.

**Methods:** A cross-sectional study was conducted to evaluate the change of CD4CD69 in MHD patients and healthy controls in our hospital from September 2015 to May 2016. A total of 164 MHD patients and 24 healthy controls were included according to the criteria. Univariate and multivariate logistic regression models after correlation analysis were executed to discover the related factors of CD4CD69 T-cell posterior to the division of the CD4CD69 T cell according to its median.

**Results:** The lymphocytes were lower, but the percentage of CD4CD69 T cells was higher in MHD patients compared with healthy controls, even after the propensity score matching based on age and sex. The percentage of CD4 T cells showed no significant difference between the two groups. Further multivariate logistic regression models revealed that CD4CD69 T cell was independently associated with serum total protein (OR 95%CI: 0.830[0.696, 0.990], *p* = .038), transferrin (OR 95%CI: 3.072[1.131, 8.342], *p* = .028) and magnesium (OR 95%CI: 16.960[1.030, 279.275], *p* = .048).

**Conclusion:** The percentage of CD4CD69 T cells, activated CD4 T cells, elevated in hemodialysis patients despite the decrease in lymphocytes. The elevated CD4CD69 T cells were independently associated with serum total protein negatively, but transferrin and magnesium positively. Strengthening nutrition, reducing the concentration of transferrin and magnesium might be beneficial to reduce the activation of CD4 T cells and improve the outcome of MHD patients.

## Introduction

In chronic kidney diseases (CKD), decreasing renal function is strongly associated with the pro-inflammatory milieu and concomitantly impaired immune system. The uremia-related immunodeficiency, especially T-cell abnormality, has a broad clinical impact on both morbidity and mortality in patients with end-stage renal disease (ESRD) on maintained hemodialysis (MHD) [1]. MHD patients have an increased risk of cardiovascular diseases, cerebrovascular diseases and a high susceptibility to infectious diseases [2,3].

Further researches showed that the premature immunological aging of the T-cell system and naïve T cells decline occurred in uremic patients [4–7], who usually had increased risks for atherosclerotic disease [8,9], high susceptibility for infectious disease [10], poor response to vaccination [11,12] and high risks of cancers [13,14]. Moreover, CD4 T cell and CD8 T cell contribute to the major parts of T cell, the former CD4 helper T cell accounting for humoral, cell immunity and inflammation, while the latter CD8 cytotoxic T responsible for killing infected cells [15]. Subsequently, Betjes MG discovered that CD4 T-cell subset can be used as a cardiovascular risk factor [8,9], and further research showed that the activated T cells might be involved in the atherosclerosis [1,16]. Therefore, regulating the activation of CD4 T cells may be an effective way to alleviate the burden of relative complications in MHD patients.

Although it is well established that CD4 T cells might contribute to the immune disturbance in MHD patients, appropriate markers to estimate the immunologic function has not been fully investigated clinically yet. Compared with CD25 and CD71, CD69 is the earliest cell surface markers expressed by T cells when activated [17]. It may act as the costimulatory molecule for T-cell activation, proliferation and differentiation [17,18]. In patients infected with HIV, CD69 can be a reliable functional marker for peripheral blood monocyte cell (PBMC) [19], and the reduction of CD69 usually means effectivity of the antiretroviral treatment [20]. In addition, CD69 can also be a useful marker to identify drug-reactive T cells in drug hypersensitivity reactions [21]. Hence, CD69 can be a sensitive marker indicating that CD4 T cell is activated and works. However, there exists no study, which focuses on restricting the activation of the CD4 T cells to improve the dialysis quality.

In our study, CD4CD69 T cells are used to evaluate the CD4 T-cell activation and potential reliable factors are explored to regulate the activation of CD4 T cells to improve the outcome of MHD patients.

## Methods

### Study population

A total of 164 patients on stable MHD at least 3 months and 24 healthy controls were recruited to the study. Patients with a history of tumors, hematopoietic disease, acute intercurrent illness, kidney transplantation, chronic infectious diseases such as hepatitis B and those who have received immunosuppressive drugs were excluded. All patients on dialysis are prescribed for three times 4-h low flux hemodialysis procedure with polysulfone membrane filter. All participants were older than 18 years old and signed the informed consent prior to the enrollment in the study. The study protocol was approved by Medical Ethics Committee of ZhongShan Hospital of Fudan University (Shanghai, China).

### Cells preparation

Whole blood was collected after the achievement of the cannulation of the vascular access but prior to the initiation of the dialysis. The blood samples were obtained with a needle and stored at the heparin tubules (BD Biosciences, San Diego, CA). Subsequently, the blood was lysed with RBC lysis buffer (155 mM NH_4_Cl and 10 mM KHCO_3_ from Shenggong Biotech, 0.1 mM EDTA from Thermofisher Scientific, pH = 7.2) at room temperature, and finally, the cells were washed twice and resuspended with staining buffer (PBS, 0.09% NaN_3_ from Sigma, 0.2% BSA from Sigma, St Louis, MO).

### Flow cytometry analysis

Cells in staining buffer were obtained and stained for 30 min at 4 °C with the following fluorescein-conjugated monoclonal antibodies: human anti-CD3-PE (Biolegend, San Diego, CA), anti-CD4-APC (eBioscience, San Diego, CA), and anti-CD69-FITC (Miltenyi Biotech, Bergisch Gladbach, Germany). Then, the cells were analyzed on BD LSR Fortessa^TM^ cell analyzer (BD Bioscience). The data analysis was carried out with Flowjo v10.1 Software (Tree Star, Ashland, OR).

### Data collection

The clinical data of included patients were recorded from the initiation of the regular hemodialysis to the year of 2016. Heart failure was diagnosed according to the ESC Guidelines for the diagnosis and treatment of acute and chronic heart failure 2012 [22]. After excluding the disorders caused by transient electrolyte imbalance, more than twice abnormal heart rhythm over a two-week interval was diagnosed as arrhythmia. The MHD-related coronary heart disease, angina pectoris, cardiac infarction and heart failure were also recorded as events of cardiovascular disease (CVD). In addition, dialysis duration, vascular access and body mass index (BMI) were also acquired, and the clinical laboratory data were obtained from the medical history system. The data about ultrasonic cardiogram were acquired with GE Healthcare (Vivid S6, Piscataway, NJ). Other related information was obtained from an electronic medical records system.

### Statistical analysis

The categorical data were expressed as frequencies and compared by using χ^2^-test. The continuous data with a Gaussian distribution were expressed as mean ± standard deviation and analyzed by using an independent samples *t*-test, while the Mann–Whitney *U*-test was used for nonparametric continuous data expressing as median (interquartile range, IQR) where appropriate. To diminish the bias, a group was extracted from the MHD group through propensity score matching with matching tolerance at 0.05 to compare with the healthy controls based on the covariates age and gender. Pearson or Spearman correlation analysis was carried out to explore the related factors of the immune cell markers for MHD group to uncover the potential relationships. The CD4CD69 T cells were divided into two layers according to the median, and univariate and multivariate logistic regression models were conducted to identify potential independent factors.

All statistical tests were two-sided and a *p* value less than .05 was considered to be significantly different. The statistical analysis was performed using SPSS v22.0 software (Chicago, IL).

## Results

### Clinical characteristics

Demographic and clinical characteristics of the hemodialysis patients (MHD group) and healthy controls (control group) are described in Table 1. Participants in MHD group are more likely to be male than healthy controls on the whole. There were nearly seven times as many persons in the MHD group than healthy controls group. Meanwhile, they were much older. As expected, in the MHD group, serum creatinine (SCr) and blood urine nitrogen (BUN) were markedly higher than that in the control group. Comparing with controls, the hemoglobin and album of the dialysis patients greatly declined. However, the globin slightly elevated. The phosphorus (P) and magnesium (Mg) accumulated in these dialysis patients, while the concentration of calcium (Ca) showed no difference between two groups. In the ultrasonic cardiogram, left ventricular ejection fraction (LVEF) was significantly lower, whereas pulmonary artery systolic pressure (PASP) was higher in dialysis patients than the corresponding values observed in the healthy controls. The median *spK*_t_*/V* value was 1.3, meaning the adequacy of the hemodialysis. Other detailed results were also listed in Table 1.

**Table 1.** Demographic and characteristics of hemodialysis patients and healthy controls.

	MHD patients (*n* = 164)	Healthy controls (*n* = 24)	*p* value
Male, *n* (%)	104 (63.4)	10 (41.7)	.042
Age, yr	62.9 ± 13.4	54.6 ± 6.1	.000
BMI, kg/m^2^	23.4 ± 3.1	24.2 ± 3.7	.305
Primary renal disease			
Chronic glomerular nephritis, *n* (%)	35 (21.3)		
Hypertensive nephropathy, *n* (%)	3 (1.8)		
Diabetic nephropathy, *n* (%)	34 (20.7)		
Lupus nephritis, *n* (%)	2 (1.2)		
Polycystic kidney, *n* (%)	14 (8.5)		
Nephrolith, *n* (%)	5 (3.0)		
Unknown, *n* (%)	71 (43.3)		
Catheter (AVF), *n* (%)	112 (68.7)		
Hemodialysis durations, months	55.0 (39.8–78.8)		
Complications			
Cardiovascular disease, *n* (%)	61 (37.4)		
Heart failure, *n* (%)	39 (23.8)		
Arrhythmia, *n* (%)	22 (13.5)		
Diabetes, *n* (%)	59 (36.0)		
Laboratory parameters			
Hemoglobin, g/L	114.0 (105.0–123.0)	138.5 (124.5–148.8)	.000
WBC, ×10^9^/L	6.3 ± 1.6	5.4 ± 1.4	.014
Neutrophil, ×10^9^/L	4.3 ± 1.5	3.1 ± 1.2	.000
Lymphocyte, ×10^9^/L	1.3 (1.0–1.6)	2.1 (1.7–2.4)	.000
Monocyte, ×10^9^/L	0.40 (0.32–0.52)	0.37 (0.28–0.43)	.174
PLT, ×10^9^/L	192.0 (156.0–231.5)	207.0 (164.0–249.3)	.183
Glucose, mmol/L	7.0 (5.7–9.1)	4.9 (4.6–5.2)	.000
TP, g/L	67.0 (64.0–70.0)	65.5 (63.0–75.0)	.167
Albumin, g/L	38.0 (36.0–40.0)	43.0 (41.0–44.8)	.000
Globulin, g/L	29.0 (27.0–32.0)	23.0 (20.3–27.5)	.000
ALT, U/L	5.0 (5.0–9.0)	22.0 (14.3–23.0)	.000
AST, U/L	15.0 (12.0–21.5)	21.0 (15.2–22.8)	.010
SCr, μmol/L	1014.6 ± 254.6	71.0 ± 14.6	.000
BUN, mmol/L	25.0 ± 5.3	5.3 ± 1.2	.000
SUA, μmol/L	420.0 (367.0–492.0)	344.0 (263.0–383.5)	.000
Ca, mmol/L	2.3 ± 0.2	2.2 ± 0.1	.292
P, mmol/L	2.2 ± 0.8	1.2 ± 0.2	.000
Mg, mmol/L	1.13 (1.05–1.22)	0.88 (0.82–0.90)	.000
β_2_-MG, mg/L	39.5 ± 9.7		
spKt/V	1.3 (1.1–1.6)		
Ferritin, ng/mL	184.0 (93.6–426.4)		
TRF, g/L	1.8 (1.6–2.0)		
NT-proBNP, pg/mL	3216.0 (1468.8–6788.0)		
hsCRP, mg/L	4.4 (1.5–9.7)		
Ultrasonic cardiogram			
PASP, mmHg	33.0 (30.0–38.3)	29.0 (25.0–32.0)	.000
LVEF, %	64.5 (61.3–67.8)	67.0 (64.0–70.0)	.002
Valvular calcification, *n* (%)	76 (47.5)	0 (0.0)	.000
Carotid plaque, *n* (%)	93 (78.8)	0 (0.0)	.000

ALT: alanine aminotransferase; AST: aspartate aminotransferase; AVF: arteriovenous fistula; BMI: body mass index; BUN: serum blood urine nitrogen; Ca: serum calcium; Glu: serum glucose; hsCRP: high sensitivity C-reactive protein; LVEF: left ventricular ejection fraction; MHD: maintained hemodialysis; Mg: serum magnesium; NT-proBNP: N-terminal pro-B-type natriuretic peptide; P: serum phosphorus; PASP: pulmonary artery systolic pressure; SCr: serum creatinine; SUA: serum uric acid; TP: total protein; TRF: transferrin; β2-MG: serum beta 2 microglobulin; WBC: white blood cells.

**Figure 1.**
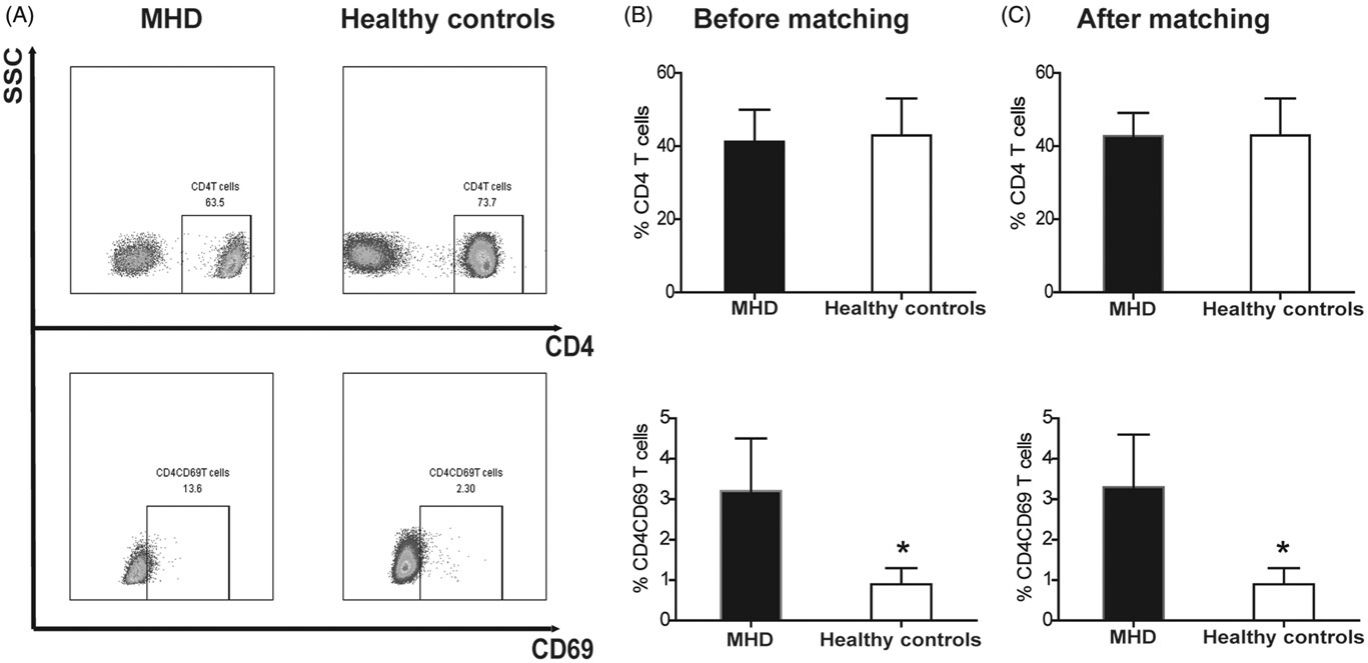
The distribution of antigen CD4 and CD69 between the MHD group and healthy controls. (A) the CD4 T cells gated on CD3 T cells and the expression of CD69 in two groups; (B)-(C) the expression of CD4 and CD4CD69 on T cells before and after propensity score matching. *p < .001.

Importantly, the quantity of the lymphocytes was significantly lower (median 1.3, IQR [1.0–1.6] versus median 2.1, IQR [1.7–2.4], *p* = .000), whereas the percentage of CD4CD69 T cells (3.2 ± 1.3 vs. 0.9 ± 0.4, *p=* .000) was significantly higher in MHD group than that in the controls. However, the percentage of CD4 T cells showed no difference between MHD and controls groups (41.2 ± 8.7 vs. 42.9 ± 10.1, *p* = .338) (Figure 1).

### Propensity score matching

The propensity score matching was executed to reduce the bias of age and gender between hemodialysis group and control group. There were no significant differences in age and gender between the two groups, indicating successful matching. After the adjustment, the basic laboratory result and the ultrasonic cardiogram were similar to the former (Table 2). And the difference between CD4CD69 T cells (3.3 ± 1.3 vs. 0.9 ± 0.4, *p <* .001) and lymphocytes (median 1.5, IQR [1.1–2.0] vs. median 2.1, IQR [1.7–2.4], *p* = .001) still remained, and the difference of the percentage of CD4 T cells (42.8 ± 6.3 vs. 42.9 ± 10.1, *p =* .973) did not exist as well, between MHD group and healthy controls (Figure 1).

**Table 2.** Characteristics of hemodialysis patients and healthy controls after the propensity score matching based on age and gender.

	MHD patients (*n* = 24)	Healthy controls (*n* = 24)	*p* value
Male, %	8 (33.3)	10 (41.7)	.551
Age, yr	56.3 ± 9.6	54.6 ± 6.1	.467
BMI, kg/m^2^	21.8 (21.2–26.0)	23.6 (21.3–27.1)	.491
Laboratory parameters			
Hemoglobin, g/L	115.8 ± 13.3	135 ± 17.2	.001
WBC, ×10^9^/L	6.4 ± 1.4	5.4 ± 1.4	.014
Neutrophil, ×10^9^/L	4.3 ± 1.4	3.1 ± 1.2	.002
Lymphocyte, ×10^9^/L	1.5 (1.1–2.0)	2.1 (1.7–2.4)	.001
Monocyte, ×10^9^/L	0.35 (0.28–0.44)	0.37 (0.28–0.43)	.959
PLT, ×10^9^/L	196.0 ± 54.8	208.8 ± 54.7	.423
TP, g/L	67.0 (64.0–70.0)	65.5 (63.0–68.5)	.167
SCr, μmol/L	1014.3 ± 227.8	71.0 ± 14.6	.000
BUN, mmol/L	26.0 ± 4.7	5.3 ± 1.2	.000
SUA, μmol/L	426 ± 66.1	322.9 ± 79.8	.000
Glucose, mmol/L	6.75 (5.5–9.1)	4.9 (4.6–5.2)	.000
Ca, mmol/L	2.35 (2.21–2.46)	2.26 (2.21–2.33)	.149
P, mmol/L	2.3 ± 0.5	1.2 ± 0.2	.000
Mg, mmol/L	1.14 (1.05–1.31)	0.88 (0.82–0.90)	.000
β_2_-MG, mg/L	39.4 ± 9.5		
spKt/V	1.3 (1.1–1.5)		
NT-proBNP, pg/mL	3339.0 (1466.3–6830.0)		
Ferritin, ng/mL	184.0 (91.7–427.4)		
TRF, g/L	1.8 (1.6–2.0)		
hsCRP, mg/L	4.3 (1.4–9.6)		
Ultrasonic cardiogram			
PASP, mmHg	30.5 (28.0–35.8)	29.0 (25.0–32.0)	.107
LVEF, %	65.5 (59.3–67.8)	68.0 (64.0–70.0)	.050

BMI: body mass index; BUN: serum blood urine nitrogen; Ca: serum calcium; hsCRP: high sensitivity C-reactive protein; LVEF: left ventricular ejection fraction; Mg: serum magnesium; NT-proBNP: N-terminal pro-B-type natriuretic peptide; P: serum phosphorus; PASP: pulmonary artery systolic pressure; SCr: serum creatinine; SUA: serum uric acid; TP: total protein; TRF: transferrin; β_2_-MG: serum beta 2 microglobulin; WBC: white blood cells.

### Correlation analysis

Correlation analysis was depicted in Table 3. With the growth of the age, both CD4 and CD4CD69 T cells decreased (*r* = −0.217, *p* = .006; *r* = −0.173, *p* = .029, respectively). However, gender, hemodialysis, body mass index (BMI) and hemodialysis durations expressed no relation with them. Patients with an arteriovenous fistula (AVF) usually had a lower percentage of CD4CD69 T cells (*r* = −0.16, *p* = .045), but the catheter was not associated with CD4 T cells (*r* = −0.127, *p* = .113). Furthermore, patients who had valvular calcification had a lower percentage of CD4CD69 T cells (*r* = −0.203, *p* = .011), but both CD4 T cells and CD4CD69 T cells were not associated with carotid plaque. Normally, CD4CD69 was not associated with heart failure (*r* = −0.110, *p* = .170), but they were significantly associated when CD4CD69 T cell was layering (*r* = −0.178, *p =* .025). Moreover, CD4CD69 T cell was associated with arrhythmia as well (*r* = −0.225, *p* = .004). The hemoglobin and lymphocytes were not associated with the CD4CD69 T cells, whereas the globin (G), transferrin and magnesium (Mg) were significantly related to CD4CD69 T cells (*r* = −0.191, *p* = .018; *r* = 0.199, *p* = .014; *r* = 0.184, *p* = .023, respectively). Moreover, the total protein (TP) showed a marginal relation with CD4CD69 T cell (*r* = −0.051, *p* = .061).

**Table 3.** Correlation analysis about CD4 and CD4CD69 T cells.

	CD4 T cell		CD4CD69 T cell	
	*r*	*p*	*r*	*p*
Age	−0.217	.006	−0.173	.029
Gender	0.04	.62	0.113	.156
HD durations	−0.031	.697	−0.18	.117
BMI	−0.079	.493	0.179	.118
Catheter	−0.127	.113	−0.16	.045
Cardiovascular disease	−0.021	.793	−0.117	.135
Heart failure	−0.086	.284	−0.110	*.170*^#^
Arrhythmia	−0.097	.227	−0.226	.004
Diabetes mellitus	−0.095	.235	−0.087	.268
Hemoglobin	0.012	.887	0.057	.488
WBC	−0.090	.273	0.027	.744
PLT	−0.18	.026	0.164	.042
Neutrophil	−0.033	.686	0.093	.251
Lymphocyte	−0.198	.014	0.03	.714
Monocytes	−0.095	.241	−0.06	.463
TP	−0.097	.23	−0.151	.061
Albumin	−0.076	.351	0.096	.236
Globulin	−0.036	.657	−0.191	.018
Glucose	−0.101	.218	−0.057	.480
BUN	0.098	.233	0.164	.044
SCr	0.138	.091	0.116	.15
SUA	0.041	.62	0.136	.095
Ca	−0.134	.102	0.114	.163
P	0.143	.081	0.100	.220
Mg	0.007	.932	0.184	.023
β2-MG	−0.039	.638	−0.084	.304
NT-proBNP	−0.121	.138	−0.228	.005
spKt/V	0.113	.193	−0.03	.729
Ferritin	−0.195	.017*	−0.098	.233
TRF	0.094	.251	0.199	.014
PASP	0.144	.079	0.025	.763
LVEF	0.128	.115	0.165	.041
Valvular calcification	−0.111	.168	−0.203	.011
Carotid plaque	−0.029	.762	−0.072	.448

# The Spearman correlation analysis shows significant difference between CD4CD69 T cell and heart failure when CD4CD69 T cells were divided into two layers according to the median (*r* = −0.178, *p* = .025) but CD4 T cells do not.

ALT: alanine aminotransferase; AST: aspartate aminotransferase; AVF: arteriovenous fistula; BMI: body mass index; BUN: serum blood urine nitrogen; Ca: serum calcium; Cr: serum creatinine; LVEF: left ventricular ejection fraction; Mg: serum magnesium; NT-proBNP: N-terminal pro-B-type natriuretic peptide; P: serum phosphorus; PASP: pulmonary artery systolic pressure; TP: total protein; TRF: transferrin; SUA: serum uric acid; β2-MG: serum beta 2 microglobulin; WBC: white blood cells.

### Regression analysis

The logistic regression models were listed in Table 4. In the univariate logistic analysis, age, gender, catheter, PLT, BUN and SUA were not associated with CD4CD69 T cells. The CD4CD69 T cell was negatively related to total protein (OR 95%CI: 0.909[0.842–0.982], *p* = .016), while positively associated with magnesium (OR 95%CI: 7.646[0.768–76.101], *p* = .083) and transferrin (OR 95%CI: 2.526[1.079–5.910], *p* = .033). The CD4CD69 T cell was associated with heart failure, arrhythmia, globin, LVEF and valvular calcification as well.

**Table 4.** Multivariate analysis of CD4CD69 T cell.

	Univariate analysis		Multivariate analysis	
	OR (95%CI)	*p* value	OR (95%CI)	*p* value
Age	0.989 (0.966–1.012)	.353	1.001 (0.965–1.038)	.970
Gender	1.317 (0.686–2.525)	.408	1.091 (0.483–2.465)	.835
Catheter	0.664 (0.334–1.322)	.244		
Heart failure	0.425 (0.199–0.910)	.028	0.912 (0.329–2.532)	.860
Arrhythmia	0.351 (0.128–0.958)	.041	0.539 (0.158–1.834)	.322
PLT	1.003 (0.998–1.008)	.254		
TP	0.909 (0.842–0.982)	.016	0.830 (0.696–0.990)	.038
Globulin	0.892 (0.817–0.973)	.010	1.004 (0.842–1.198)	.963
BUN	1.022 (0.962–1.084)	.485		
SUA	1.003 (0.999–1.007)	.171		
Mg	7.646 (0.768–76.101)	.083	16.960 (1.030–279.275)	.048
NT-proBNP	1.000 (1.000–1.000)	.010		
TRF	2.526 (1.079–5.910)	.033	3.072 (1.131–8.342)	.028
LVEF	1.060 (1.006–1.115)	.027	1.003 (0.935–1.076)	.937
Valvular calcification	0.566 (0.300–1.068)	.079	0.715 (0.276–1.850)	.489

BUN: serum blood urine nitrogen; LVEF: left ventricular ejection fraction; Mg: serum magnesium; NT-proBNP: N-terminal pro-B-type natriuretic peptide; SUA: serum uric acid; TP: total protein; TRF: transferrin.

In multivariate logistic regression analysis, the CD4CD69 T cell showed negative relationships with total protein (OR 95%CI: 0.830[0.696–0.990], *p* = 0.038) and positive relation with magnesium (OR 95%CI: 16.960[1.030–79.275], *p* = .048) and transferrin (OR 95%CI: 3.072[1.131–8.342], *p* = .028).

## Discussion

In our study, we found that the percent of CD4CD69 T cell elevated in the MHD patients, and it was independently negatively associated with total protein, and positively associated with magnesium and transferrin.

MHD patients showed an impaired immune function because of the declined number of lymphocytes, compared with healthy people [7]. The decreased number of CD4 T cells also threatened the health of patients [23]. But in our study, the percentage of CD4 T cells was not different between healthy controls and hemodialysis patients, even adjusted for age and gender, which was supported by another research [24]. However, the percentage of CD4CD69 T cells elevated significantly in hemodialysis patients, and they were more susceptible to apoptosis [1]. The uremic and inflammatory microenvironment may play a major role in this process [25,26], especially considering the CD4 helper T cell just accounting for inflammation. On the surface of the T cells, CD69 was an antigen representing the initial activity of T cell [18]. So the activated T cells, CD4CD69 T cells, may secrete cytokines such as IL-8 to contribute to this immune disturbance [26].

Importantly, inflammation and abnormally activated CD4 T cells may be associated with the hemodialysis complications. CD4 T-cell abnormality was associated with the cardiovascular disease in ESRD patients [9]. We found that CD4CD69 T cells were associated with heart failure and arrhythmia, but not independently. Activated CD4 T-cell subsets are associated with the progression of heart failure [27,28], and the activation of peripheral blood CD3 T lymphocytes is associated with atrial fibrillation [29]. But we found higher morbidity rate of some CVD events was negatively associated with CD4CD69 T cells. It is whether the intervention of uremic environment or the unexisted follow-up or the limited patients that cause the phenomenon, which is still unknown. Both valvular calcification and carotid plaque are important risk factors for CVD [30,31], and T cell would regionally accumulate and activate in the atherosclerotic plaque [33–35]. Further research showed CD4 T-cell recruitment in atherosclerotic plaque was induced by IL-12 [36] and this activated inflammatory cell was associated with the rupture of the plaque [37]. However, we do not find the significant difference between the CD4CD69 and carotid plaque; further investigation may be needed to confirm the reason, but regulating the activation of CD4 T cells may be an effective way to prevent the cardiovascular disease in hemodialysis patients and seeking effective reliable method is of great clinical significance.

Moreover, we also found that CD4CD69 T cells were independently associated with the total protein, transferrin and magnesium. The serum total protein could be used to evaluate the nutrition status of patients [38]. The malnutrition would impair the immune system and increase the susceptibility to infectious disease [39]. The MHD patients in poor nutrition might benefit from the improved nutrition status. But there is rare research about the relation between serum total protein and CD4 T cell–mediated inflammation for the moment. Transferrin, conveying the iron in the blood, is a kind of glycoprotein [40]. Transferrin, also as a growth factor, could stimulate the T lymphocytes to proliferate and express CD69 [41]. Thus, higher transferrin would activate more T cells and impair the immune system. Magnesium is an essential ion participating in energy metabolism and protein synthesis in the human body. Moreover, the magnesium transporter protein 1 could be a new intermediate in TCR signaling, which is linked to T-cell deficiency when mutated [42,43]. Further studies showed that magnesium would regulate the secretion of cytokine of CD4 helper T lymphocytes to change the inflammatory status of the organism [44,45]. Therefore, reducing the concentration of transferrin and magnesium may contribute to the reduction of activated CD4 T cells theoretically. However, the validity and efficiency of these methods still need further confirmation by large strict cohort studies.

**Figure 2.**
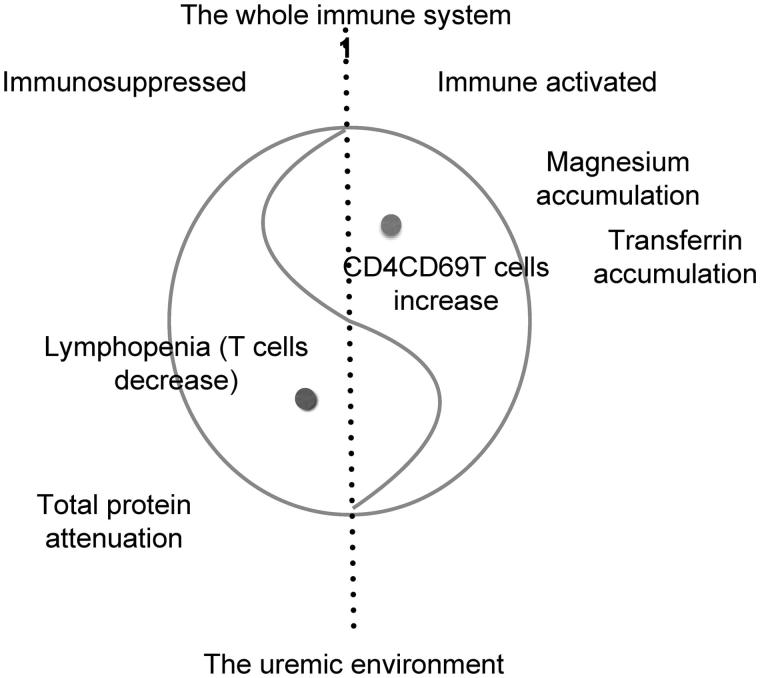
The model about the balance of the immune system. The whole immune system can be recognized as ‘1’ philosophically. The immune system reaches a curve line balance in hemodialysis patients. The accumulation of magnesium and transferrin promote the activation of immune system and inflammation, while the lymphopenia and the decreased total protein shake the cornerstone of the immune system, resulting in the immunosuppression.

As a summary, a model of immune balance in hemodialysis was introduced in Figure 2. The left side of the dotted line represents immunosuppressed, and the other hand is immune activated. Moreover, the whole circle represents the entire immune system ‘1’. As for the healthy controls, the whole system is steady and balanced, so the dotted line stands in the central of the circle, and the heads of two ‘fishes’ should be slight. In the uremic environment, lymphopenia and the calculated absolute number of CD4 T cells lie on the left, while the activated CD4 T cells hold the position of the right. When the uremic environment exists, two fishes would like to swim to the other side, leading to the bigger ‘fish’ heads, until the new curved line balance (relative balance) takes shape. So the immunosuppression and immune hyperfunction coexist in uremic patients, which seems to be consistent with most immune cells [1], expressing a higher percentage of activated cells but a lower absolute number of total cells. Usually, once this new curved line balance forms, it is hard to get it back *in situ* because of some reasons such as the insufficient lymphopenia-induced proliferation (LIP) [46]. Under the new balance, some diseases or complications are easier to take place. The environment, the immune suppression and immune activation can be intervened at the same time to improve the status of patients.

In conclusion, the percentage of CD4CD69 T cells, activated CD4 T cells, elevated in hemodialysis patients despite the decrease of lymphocytes. The elevated CD4CD69 T cells were independently associated with serum total protein negatively, but transferrin and magnesium positively. Strengthening nutrition, reducing the concentration of transferrin and magnesium might be beneficial to reduce the activation of CD4 T cells and improve the outcome of MHD patients.
